# Supplementary material for: Experimental determination of evolutionary barriers to horizontal gene transfer
Source: BMC Microbiol. 2020 Oct 28;20:326. doi: 10.1186/s12866-020-01983-5 (PMC7592521; doi:10.1186/s12866-020-01983-5)
Supplement: Supplementary file 3 — Additional file 3. Supplementary figures and tables that accompany the paper. [file 12866_2020_1983_MOESM3_ESM.docx]

Supplementary Materials

###

###

***Sup. Fig. 1.*** *Verifying the expression of a subset of transferred genes at the level of translation. We cloned the GFP gene at the end of the transferred gene as translational fusions. In this setting, level of GFP expression indicates the expression level of the transferred genes. Cultures grown overnight in rich M9 medium were diluted 1000x and handled under the same conditions as the competition assays. One hour after adding the inducer (5ng/mL ATC), OD_600_ and GFP_540_ emissions were measured, and GFP_540_ readings were normalized with the corresponding OD_600_ readings, given here as the Fusion GFP expression. Background is the normalized GFP expression of the E. coli MG1655 att-λ::(tetR-Sp^R^) att-p21::(CFP-Kn^R^) recipient strain carrying the empty plasmid. Error bars represent SD of four replica experiments.*

**Sup. Fig. 2**. Diagram of the expression plasmid used in this study, modified from Lutz and Bujard (1997). The gene of interest is cloned between the restriction enzyme sites AvrII and PstI/HindIII. The empty plasmid contained only the ribosomal binding site and restriction enzymes sites between the P_LtetO-1_ promoter and the terminator T1. Amp^R^ is the resistance gene for ampicillin. The terminators T1 of the rrnB operon and t_0_ of phage lambda.

**Sup. Fig. 3.** Post-hoc power analysis performed to estimate the sensitivity of our selection coefficients (s) measurements during competition assays. ∆s ≈ 0.005 as the potential effect size of our measurements is calculated for α=0.01, β=0.80 and sd=0.008, two-tailed test. The standard deviation of this test is calculated as the average standard deviation observed during the selection coefficient calculations of all genes (including the within plate control competitions) performed in this study.

**Sup. Fig. 4**. Number of disordered regions in the amino-acid sequences of newly transferred 44 S. Typhimurium genes plotted against gene length (nucleotides). Using simple linear regression, the relationship is significant with p<0.001 (see Methods section – Statistical analyses).

***Sup. Fig. 5.*** *DFE of newly transferred 44 E. coli orthologs expressed in E. coli. On the x-axis, genes are sorted by their fitness effects (s). Error bars indicate the 95% CI of the selection coefficients from 32 replicate measurements for each gene. Empty circles represent the 2 genes with effects not significantly different from zero. Embedded plot gives the histogram representation of the same data*.

**Sup. Fig. 6**. Comparison of 36 newly transferred and deleterious S. Typhimurium genes grouped as dosage sensitive and insensitive. Dosage sensitive and insensitive genes do not differ **a.** in their mean gene length; **b.** in the mean number of disordered regions in their amino-acid sequences; and **c.** in their level of divergence.

**Sup. Fig. 7**. Selection coefficients of transferred E. coli orthologs are plotted against the fold change in their expression levels resulting from the induction of the expression plasmid during the competition assays. Horizontal red line marks the selection coefficients at s = – 0.1, and vertical red line marks the 10-fold increase in the expression of transferred genes compared to their endogenous expression under the experimental conditions, calculated using TPM data obtained through RNA-seq. Fitness effect of a transferred gene was highly deleterious only if its expression increased more than 10-fold. **Sup. Table 1.** Selection coefficients of all 44 S. Typhimurium and E. coli orthologs expressed in E. coli, sorted according to the selection coefficients of S. Typhimurium orthologs.

| **Gene Name** | **STM**  **Gene ID** | **STM**  **Sel.Coef.** | **p-value** | **ECO**  **Gene ID** | **ECO**  **Sel.Coef.** |
| --- | --- | --- | --- | --- | --- |
| *lpxD* | STM0226 | 0.00921 | **<.001** | b0179 | 0.00959 |
| *ybhK* | STM0801 | 0.00896 | **<.001** | b0780 | -0.03490 |
| *hfq* | STM4361 | 0.00285 | **.002** | b4172 | 0.00336 |
| *infC* | STM1334 | 0.00125 | .074 | b1718 | 0.00266 |
| *pnp* | STM3282 | 0.00037 | .719 | b3164 | -0.03917 |
| *rlmL* | STM1061 | 0.00035 | .326 | b0948 | -0.00506 |
| *ydiI* | STM1366 | -0.00082 | .102 | b1686 | -0.00670 |
| *sapF* | STM1696 | -0.00181 | **.003** | b1290 | -0.01514 |
| *yacL* | STM0160 | -0.00206 | **.017** | b0119 | -0.00860 |
| *exbB* | STM3159 | -0.00244 | .077 | b3006 | -0.00302 |
| *hybG* | STM3143 | -0.00283 | **.004** | b2990 | -0.01440 |
| *rplI* | STM4394 | -0.00591 | **<.001** | b4203 | -0.00038 |
| *moaE* | STM0806 | -0.00624 | **<.001** | b0785 | -0.02008 |
| *cbpA* | STM1112 | -0.00678 | **<.001** | b1000 | -0.02457 |
| *uspG* | STM0614 | -0.00681 | **<.001** | b0607 | -0.00716 |
| *rimI* | STM4558 | -0.00776 | **<.001** | b4373 | -0.00356 |
| *ridA* | STM4458 | -0.00917 | **<.001** | b4243 | -0.00369 |
| *cspE* | STM0629 | -0.00923 | **<.001** | b0623 | 0.00589 |
| *dps* | STM0831 | -0.00963 | **<.001** | b0812 | -0.00648 |
| *ibpB* | STM3808 | -0.00992 | **<.001** | b3686 | -0.00045 |
| *glyQ* | STM3656 | -0.01117 | **<.001** | b3560 | -0.41062 |
| *yibL* | STM3689 | -0.01623 | **<.001** | b3602 | -0.01602 |
| *dnaQ* | STM0264 | -0.01959 | **<.001** | b0215 | -0.04294 |
| *cspD* | STM0943 | -0.02427 | **<.001** | b0880 | -0.13406 |
| *iscS* | STM2543 | -0.02693 | **<.001** | b2530 | -0.06198 |
| *hupA* | STM4170 | -0.02744 | **<.001** | b4000 | -0.04459 |
| *kdpD* | STM0703 | -0.03411 | **<.001** | b0695 | -0.02397 |
| *clpA* | STM0945 | -0.04958 | **<.001** | b0882 | -0.02243 |
| *yqjI* | STM3215 | -0.05091 | **<.001** | b3071 | -0.01484 |
| *pstB* | STM3854 | -0.05355 | **<.001** | b3725 | -0.04937 |
| *acpP* | STM1196 | -0.07789 | **<.001** | b1094 | -0.06697 |
| *selB* | STM3682 | -0.08022 | **<.001** | b3590 | -0.10939 |
| *hupB* | STM0451 | -0.08211 | **<.001** | b0440 | -0.03703 |
| *lexA* | STM4237 | -0.10157 | **<.001** | b4043 | -0.07425 |
| *malP* | STM3514 | -0.10279 | **<.001** | b3417 | -0.13412 |
| *fadJ* | STM2388 | -0.13126 | **<.001** | b2341 | -0.02219 |
| *yadG* | STM0172 | -0.13294 | **<.001** | b0127 | -0.03153 |
| *thiI* | STM0425 | -0.15182 | **<.001** | b0423 | -0.21239 |
| *rne* | STM1185 | -0.15861 | **<.001** | b1084 | -0.22364 |
| *leuS* | STM0648 | -0.27576 | **<.001** | b0642 | -0.30644 |
| *srmB* | STM2643 | -0.34174 | **<.001** | b2576 | -0.03822 |
| *lolA* | STM0961 | -0.43262 | **<.001** | b0891 | -0.01427 |
| *topB* | STM1298 | -0.45193 | **<.001** | b1763 | -0.59428 |
| *uvrC* | STM1946 | -0.60555 | **<.001** | b1913 | -0.59745 |

*p-values are for the fitness effects of S. Typhimurium orthologs being different from zero. Fields indicated with* ***bold*** *are significant values, α = 0.05 after corrections for multiple testing with BH-FDR method* (59)*.*

**Sup. Table 2.** Data used in the multiple linear regression analysis, related to the transferred S. Typhimurium genes.

| STM  Gene ID | Functional  Category | # PPI | Deviation  %GC | Deviation  F_OP_ | Gene  Length | HT / Core | I/O | Disordered  regions | Dosage |
| --- | --- | --- | --- | --- | --- | --- | --- | --- | --- |
| STM0160 | Unknown | 33 | 0.001 | 0.058 | 363 | NA | U | 3 | Sensitive |
| STM0172 | Operational | 39 | 0.002 | 0.016 | 927 | HT | O | 5 | Insensitive |
| STM0226 | Operational | 30 | 0.008 | 0.041 | 1026 | Core | O | 2 | - |
| STM0264 | Informational | 12 | 0.010 | 0.020 | 732 | Core | I | 6 | Sensitive |
| STM0425 | Informational | 1 | 0.002 | 0.012 | 1449 | HT | I | 5 | Sensitive |
| STM0451 | Informational | 20 | 0.005 | 0.000 | 273 | Core | I | 8 | Insensitive |
| STM0614 | Operational | 12 | 0.022 | 0.028 | 429 | HT | O | 2 | Sensitive |
| STM0629 | Informational | 31 | 0.002 | 0.071 | 210 | Core | I | 2 | Insensitive |
| STM0648 | Informational | 8 | 0.025 | 0.043 | 2583 | Core | I | 5 | Sensitive |
| STM0703 | Operational | 1 | 0.013 | 0.006 | 2685 | Core | O | 6 | Insensitive |
| STM0801 | Operational | 3 | 0.030 | 0.073 | 909 | Core | O | 0 | - |
| STM0806 | Operational | 0 | 0.043 | 0.033 | 453 | HT | O | 1 | Sensitive |
| STM0831 | Operational | 12 | 0.007 | 0.018 | 504 | Core | O | 4 | Insensitive |
| STM0943 | Informational | 28 | 0.036 | 0.006 | 222 | Core | I | 1 | Sensitive |
| STM0945 | Informational | 31 | 0.016 | 0.037 | 2277 | Core | I | 4 | Insensitive |
| STM0961 | Operational | 1 | 0.005 | 0.017 | 615 | Core | O | 3 | Insensitive |
| STM1061 | Informational | 21 | 0.008 | 0.011 | 2109 | Core | I | 4 | - |
| STM1112 | Informational | 23 | 0.006 | 0.104 | 921 | Core | I | 6 | Sensitive |
| STM1185 | Informational | 35 | 0.028 | 0.063 | 3204 | Core | I | 0 | Sensitive |
| STM1196 | Operational | 20 | 0.002 | 0.000 | 237 | Core | O | 3 | Insensitive |
| STM1298 | Informational | 16 | 0.012 | 0.022 | 1950 | Core | I | 2 | Sensitive |
| STM1334 | Informational | 36 | 0.010 | 0.006 | 543 | Core | I | 1 | - |
| STM1366 | Operational | 25 | 0.052 | 0.022 | 411 | Core | O | 0 | - |
| STM1696 | Operational | 2 | 0.032 | 0.011 | 807 | Core | O | 4 | Sensitive |
| STM1946 | Informational | 21 | 0.017 | 0.008 | 1833 | Core | I | 4 | Insensitive |
| STM2388 | Operational | 3 | 0.017 | 0.045 | 2148 | Core | O | 0 | Insensitive |
| STM2543 | Operational | 5 | 0.016 | 0.064 | 1215 | Core | O | 2 | Sensitive |
| STM2643 | Informational | 31 | 0.004 | 0.027 | 1335 | Core | I | 2 | Insensitive |
| STM3143 | Operational | 7 | 0.006 | 0.048 | 249 | Core | O | 1 | Sensitive |
| STM3159 | Operational | 14 | 0.027 | 0.012 | 735 | Core | O | 0 | - |
| STM3215 | Informational | 14 | 0.001 | 0.031 | 648 | HT | I | 1 | Insensitive |
| STM3282 | Informational | 37 | 0.022 | 0.008 | 2136 | Core | I | 0 | - |
| STM3514 | Operational | 25 | 0.031 | 0.020 | 2394 | Core | O | 1 | Sensitive |
| STM3656 | Informational | 9 | 0.003 | 0.059 | 912 | Core | I | 0 | Sensitive |
| STM3682 | Informational | 29 | 0.008 | 0.039 | 1851 | Core | I | 0 | Sensitive |
| STM3689 | Informational | 24 | 0.001 | 0.058 | 363 | NA | I | 0 | Sensitive |
| STM3808 | Operational | 3 | 0.029 | 0.119 | 429 | NA | O | 1 | Insensitive |
| STM3854 | Operational | 17 | 0.001 | 0.027 | 774 | Core | O | 2 | Insensitive |
| STM4170 | Informational | 34 | 0.006 | 0.011 | 273 | Core | I | 8 | Sensitive |
| STM4237 | Informational | 14 | 0.032 | 0.005 | 609 | Core | I | 5 | Insensitive |
| STM4361 | Informational | 24 | 0.011 | 0.029 | 309 | Core | I | 0 | - |
| STM4394 | Informational | 27 | 0.012 | 0.013 | 450 | Core | I | 2 | Insensitive |
| STM4458 | Operational | 9 | 0.017 | 0.031 | 387 | Core | O | 4 | Insensitive |
| STM4558 | Informational | 12 | 0.027 | 0.027 | 447 | Core | I | 3 | Insensitive |

*The data is curated using Hu et al. (26) for # PPI, EcoGene (ecogene.org,* (54)*) for horizontally transferred or core (HT/Core), Gene Ontology and MultiFun annotations (25) for Informational/Operational (I/O) and Globplot (globplot.embl.de,* (36) *for Disordered regions.*

**Sup. Table 3.** Result of the multiple regression analysis, F_5,37_ = 2.24.

|  | R^2^ | B | SE’B | beta | *p-*value |
| --- | --- | --- | --- | --- | --- |
|  |  |  |  |  |  |
| Model | 0.232 | - | - | - | 0.071 |
|  |  |  |  |  |  |
| Intercept |  | -0.136 | 0.070 | - | 0.059 |
|  |  |  |  |  |  |
| Functional Category |  | 0.043 | 0.045 | 0.155 | 0.354 |
|  |  |  |  |  |  |
| Protein Interactions |  | 2.214e^-03^ | 1.874e^-03^ | 0.191 | 0.245 |
|  |  |  |  |  |  |
| Deviation in F_OP_ |  | 1.054 | 0.758 | 0.203 | 0.173 |
|  |  |  |  |  |  |
| Deviation in %GC |  | 1.635 | 1.638 | 0.150 | 0.325 |
|  |  |  |  |  |  |
| Gene Length |  | -6.278e^-05^ | 2.489e^-05^ | -0.370 | 0.016 |
